# Virtualization of Event Sources in Wireless Sensor Networks for the Internet of Things

**DOI:** 10.3390/s141222737

**Published:** 2014-12-01

**Authors:** Néstor Lucas Martínez, José-Fernán Martínez, Vicente Hernández Díaz

**Affiliations:** Centro de Investigación en Tecnologías Software y Sistemas Multimedia para la Sostenibilidad (CITSEM), Universidad Politécnica de Madrid (UPM), Edificio La Arboleda, Campus Sur, Carretera de Valencia km.7, Madrid 28031, Spain; E-Mails: jf.martinez@upm.es (J.-F.M.); vicente.hernandez@upm.es (V.H.D.)

**Keywords:** wireless sensor networks, internet of things, event-driven, virtualization

## Abstract

Wireless Sensor Networks (WSNs) are generally used to collect information from the environment. The gathered data are delivered mainly to sinks or gateways that become the endpoints where applications can retrieve and process such data. However, applications would also expect from a WSN an event-driven operational model, so that they can be notified whenever occur some specific environmental changes instead of continuously analyzing the data provided periodically. In either operational model, WSNs represent a collection of interconnected objects, as outlined by the Internet of Things. Additionally, in order to fulfill the Internet of Things principles, Wireless Sensor Networks must have a virtual representation that allows indirect access to their resources, a model that should also include the virtualization of event sources in a WSN. Thus, in this paper a model for a virtual representation of event sources in a WSN is proposed. They are modeled as internet resources that are accessible by any internet application, following an Internet of Things approach. The model has been tested in a real implementation where a WSN has been deployed in an open neighborhood environment. Different event sources have been identified in the proposed scenario, and they have been represented following the proposed model.

## 1. Introduction

The Internet of Things paradigm [1] aims at supporting *smart object* connectivity so that any physical object (home appliances, cars, products in a mall, smartphones, *etc.*) can interact with each other in an unmanned-wise manner and provide humans with a better daily experience. Existing technologies like WSN or RFID, among others, are envisioned as foundation technologies for the IoT. To accomplish that task, standards are required, as they will encourage interoperability among devices and solutions from different stakeholders. Several international standardization bodies have increased their activities in the IoT area, as [2] reports.

Furthermore, research projects are also heavily involved in specifying and describing reference models, layered architectures, information models, ontologies and so forth, that could be adopted in the near future by the IoT standardization bodies, as well as by the solutions providers. The IoT-A European research project [3] is specifying an architectural reference model [4] that will provide a common framework for IoT-A solutions, overcoming interoperability challenges. Briefly, the proposed model in [4] virtualizes devices to obtain their computational representation. Virtualizing device capabilities provides significant improvements to software architectures as they become decoupled from the specific (proprietary) way the hardware resources are accessed. The virtual representation of the resources provided by a set of devices enables applications from different domains to access seamlessly the same capabilities supported by a shared, heterogeneous hardware infrastructure. Thus, the deployment and maintenance costs of new solutions are reduced. No new duplicating hardware infrastructure is needed, as the already existing solutions are rapidly reused for almost any upcoming application domain. Upgrading systems with new more powerful devices is not likely to impact on the running software solutions, as long as the new devices are virtualized in the same way [5,6].

The so-called *virtual device* in IoT-A exposes its semantically described resources to any other actor by means of services, accomplishing a Service Oriented Architecture (SOA) approach [7]. Different SOA technologies are being used and worldwide accepted, but the one becoming preferred for its simplicity and low overload whenever constrained devices are involved is RESTful Web Services, based on the Representational State Transfer (REST) approach [8]. In a Resource Oriented Architecture (ROA) solution, system entities can only create, read, update and delete resources hosted by any other system entity.

To save network resources WSN-based solutions are usually event-driven systems. Sensor nodes go into sleep mode until a significant event is triggered, notifying subscribers about it only when necessary. This behavior reduces the energy consumption and the number of messages exchanged across the network, improving the overall performance of the WSN. The design of such systems has to comply with IoT reference models in order to be integrated in any IoT solution. Therefore, the elements in an event-driven system like in a WSN have to be properly modeled to match the REST approach, along with the IoT reference model (e.g., IoT-A).

This paper proposes a model that enables REST compliance of event-driven systems, virtualizing the corresponding devices as it is being proposed in IoT reference models like IoT-A. The model has been developed and successfully integrated in a pilot in Web of Objects (WoO), a European research project included in the ITEA2 research program and funded by the Spanish Ministry for Industry, Energy and Tourism. WoO aims at providing a model to develop a Web of Objects, comprised of objects in an IoT environment that cooperate smartly to arrange and provide a web of services and complex virtual devices.

The different elements of the models have been integrated in nano Service Oriented Middleware (nSOM), a middleware architecture that is being developed by the Technical University of Madrid (UPM) for deploying WSN solutions on any hardware platform.

The event sources in the WSN are registered and published in a repository, either statically or dynamically by discovering them, as REST resources. The nSOM event manager will send a subscription message to every event-source (sensor node). Any external subscriber will also register in the nSOM repository and will be exposed as a resource. Whenever an event is triggered, the sensor node will notify the event manager that will create a new resource in the repository representing the new event and will notify the appropriate subscriber about that new resource. The event sources have only one subscriber, the event manager, thus avoiding the storage of large lists of subscribers and reducing the number of messages across the WSN when notifying subscribers. The repository and the event manager are nSOM elements running in the gateway that connects the WSN to another network.

## 2. Background

Advantages and drawbacks of WSN virtualizations are described in [5,6,9] and have been outlined in the previous section. The foundations of the WSN virtualization implementation to be detailed in the coming sections are described in the following paragraphs.

nSOM is a middleware architecture that virtualizes a WSN node capabilities so that any tiny application could be run in such nodes. Moreover, such tiny applications might virtualize new capabilities in the node that is hosting them, extending the catalog of its available functionalities. It has been designed for constrained devices and targets as objectives low memory and processor footprint utilization. The core elements in nSOM (as the service registry manager or the context manager) comply with the SOA principles, decoupling what an entity is able to do from the specific way the entity is implemented. The above mentioned tiny applications might also act as service providers and/or service consumers, interoperating with other tiny applications or nSOM core elements running in other nodes in the network. Thus, nSOM enables a WSN to provide a virtual set of SOA services for accessing nodes capabilities, hiding the physical deployment of nodes and tiny applications that actually carry the set out.

nSOM also supports the publish/subscribe messaging scheme, which is more efficient for WSNs when used in event-driven applications. In such applications, significant events are detected and triggered by monitoring entities (WSN nodes), which become events publishers, and are consumed by entities that process and provide a proper response to the specific event, which become events subscribers. This approach reduces the amount of messages throughout the network as they are generated and transmitted over the WSN only when an event is required to be triggered. Publish/subscribe paradigm utilizes an initial overhead when the publishers are announced and the subscribers subscribe to the publishers. The benefits from publish/subscribe messaging approach in WSN have been highlighted in [10]: devices and systems from different providers are readily integrated, publishers and subscribers are decoupled (all must agree on the same message format), the network is scalable and its efficiency is higher. Working with the opposite approach would imply that applications poll the monitoring entities from time to time looking for environmental changes by using a prominent network overhead, which usually is not a practical solution. The nSOM specification describes a protocol for supporting inter-node communications that includes messages for the publish/subscribe approach, similar to those in MQTT protocol as described in [10], but complying with the nSOM architecture principles.

IoT-A also invests on virtualizing a physical device by means of a double approach: creating a digital representation of the physical device (so called virtual device) that includes how to address it, how to map internal registers, *etc.*, and modeling its resources as SOA compliant services. The resources of similar devices might be modeled with the same set of services, encouraging reutilization. Furthermore, the way any application accesses a resource is decoupled from the intrinsic complexity of the device, allowing applications from different domains share the same set of devices.

REST is nowadays a worldwide accepted SOA technology that can be used to support the IoT spread. The REST model proposes that cooperating entities might share resources; it is likely that an entity offers a resource and other entities might want to operate on it (get a copy of the resource, modify it, create it, remove it, *etc.*). The set of available operations is limited and unique for all the resources. The semantic of each operation is also predefined. That is the reason why it is also said that REST can be regarded as a ROA approach. All those characteristics define an easy set of rules for entities interoperation, making REST a lightweight SOA technology. Nonetheless, the protocol stack that most of the REST implementations propose might postpone its promotion in domains supported by tiny devices without enough computational resources. To cope with that, COAP is envisioned as the protocol for transporting REST messages without wasting too many resources.

## 3. Proposal

The design of any system architecture involving the usage of a WSN implies a set of challenges that must be considered. Akyildiz [11] identified in the past various factors that have an influence in the design of the network, including fault tolerance, scalability, hardware constraints, topology and communications among others. In the case of using also an event-driven model there are two other factors, regarding whether the event notification must be guaranteed, and if affirmative, which the accepted delivery time is.

The proposal described in this section includes several innovative points. The most significant one is the way events in a WSN are modeled, as it leverages the virtualization of new node capabilities in the WSN and eases the integration of WSN in IoT infrastructures. Two different points of view are considered: the inner-WSN point of view, or how the nodes in the same WSN manage an event, and the outer-WSN point of view, or how the applications external to the WSN are notified of the triggered events and event sources.

From the inner-WSN point of view, the nodes operate in a SOA-wise manner. Traditionally, the capabilities of the nodes in a WSN have not been exposed using well known approaches, broadly accepted in the business information systems, but proprietary solutions. In this proposal, the nodes triggering events must register in a service registry in the WSN and implement a service to register subscribers. The nodes that wish to be notified of specific events must look up the service registry for the proper event publisher and subscribe to the publisher event notifications by accessing the corresponding service. Thus, new virtual event sources can be made readily available by means of service composition or orchestration techniques.

From the outer-WSN point of view, events are modeled complying with the ROA approach, similarly as proposed in [10,12–14]. In this proposal, the list of available event sources in the WSN can be retrieved as any other RESTFul resource. It is automatically updated due to the discovery protocol implemented by the components in the nSOM middleware. Each event source is described using ontologies, enhancing interoperability among different stakeholders' solutions. A hierarchical naming convention, as suggested in [10], is also proposed in this paper.

The proposed model considers specially the response time, as it is a requirement of the scenario in which it will be tested. Additionally, the constrained nature of the resources is considered, in the form of reducing the amount of required messages between the entities participating in the notification of an event when it happens in the domain of the WSN.

### 3.1. The Virtualization Model

The project IoT-A has proposed a domain model in which any resource, either constrained or not, can be mapped and represented as a virtual entity by means of the resources it exposes [4]. This is also applicable for every node that takes part in a WSN, and can also be extended to the events detected, or even generated by the sensors themselves.

Typically, the nodes of a WSN are not able to communicate directly to the Internet, or even to expose themselves by means of resources and services. The way to accomplish the interconnection between the WSN and the Internet is done by means of a gateway. The gateway is responsible for translating and routing the messages between the two networks.

**Figure 1.**
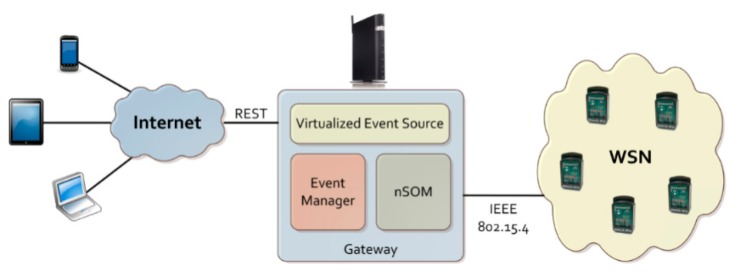
Virtualization model scenario.

In this proposal the gateway provides a virtual representation of the nodes in the network. It does that by associating a virtual entity to the exposed resources, and thus defining a set of services that give access to the resources. It can also create new resources on demand to manage the new events that are notified by the nodes in the WSN. And everything is done according to the model put forward in [4]. Figure 1 shows a graphical depiction of the proposed model.

### 3.2. Virtualization of Existing Event Sources

The basic functionality of the model can be described in three stages. In the first one the gateway proceeds to discover the nodes in the WSN, and detects if they provide events. The way the discovery is accomplished is not covered in this paper, and it can be done in several ways, usually using either the passive or the active methods that are both covered by the nSOM middleware.

**Figure 2.**
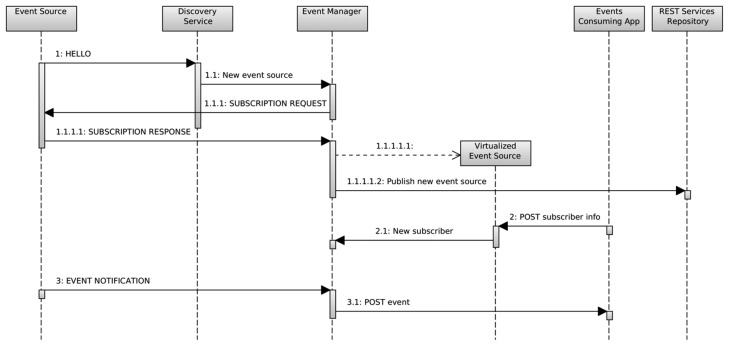
Event source virtualization and event notification and processing.

Once an event source has been detected, the event manager component in the gateway automatically subscribes itself to it as an event consumer. Afterwards, it—the event manager—exposes the event as a REST service. This REST services follow the design pattern proposed by Li [15], and corresponds to the messages labeled as 1.X in the sequence diagram shown in Figure 2.

The second stage is applied when any kind of user, either a human or another application or service, requires to be notified whenever an event source generates an event. This is done using a traditional subscriber methodology. The interested entity, once it becomes aware about the existence of a specific event source, sends a POST message to the virtualized event source, so it can register a new subscriber, or by means of REST, create a new resource representing the subscriber. All the resources managed by the proposed system are described in Table 1.

In order to be notified of an event, the subscriber must provide some callback function. In this proposal, this callback is also a REST resource managed directly by the subscriber, and linked to the resource created by the event manager to represent it. All this second stage corresponds to the messages labeled as 2.X in Figure 2.

Finally, when an event is generated in the event source, it is notified firstly to the event manager by using a lightweight message from the nSOM middleware. The event manager then processes the event and notifies every event consumer using the subscriber's callback URI to the REST resource that represents the handler for that specific event. To accomplish this purpose, a POST method is used, which means that a new event has been detected and can be interpreted as a request for the creation of the resource that handles the event. This stage corresponds to the messages labeled as 3.X in Figure 2.

All the messages transferring the information through the REST requests are JSON based. The messages interchanged in the WSN are shown in uppercase, and use a lightweight protocol specifically developed for the nSOM middleware. In Figure 2 the *Event Source* is any device in the WSN capable of generating an event. The *discovery Service*, the *Event Manager* and the *REST Services Repository* are nSOM elements running in the gateway.

### 3.3. Composing New Virtualized Event Sources

When talking about virtualization there is another approach. Islam [9] defines a Virtual Sensor Network as a “collaborative wireless sensor network” where a subset of the nodes of a WSN cooperates with other entities to accomplish a certain task. This is essentially a service composition.

Service composition can be done from the gateway, as this can be deployed on an unconstrained device capable of running complex applications. For our interest, we have explored the service composition from inside the WSN. A node, either a sensor or a simple processing unit, orchestrates the information gathered from a subset of sensors in the network, and creates a new complex service that is exposed as any other.

**Figure 3.**
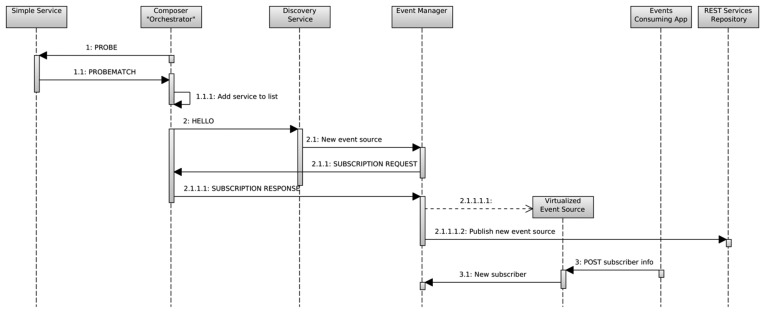
Composition of a virtual event source.

In the case of a composed service able to generate events, the sequence of actions follows the diagram of Figure 3. Here, the node responsible for the service composition, named “*Orchestrator*”, does an active discovery of the required services by broadcasting a PROBE message to the WSN. Any sensor providing that service and capable of responding to the PROBE message replies with a PROBEMATCH message addressed to the *Orchestrator*. It then checks whether the responding service is valid for the desired service composition, and if affirmative, adds it to an internal list of service providers. This corresponds to the messages labeled as 1.X in Figure 3.

Once the service has been successfully composed, it is announced to the *Discovery Service* on the gateway, and registered as a new event source in the *Event Manager*, following the same sequence of actions as with an already existing simple event source. Indeed, from the point of view of any other actor in the system besides the *Orchestrator*, it is just another event source.

The generation of an event in a composed event source is different when compared to the simple one. The *Orchestrator* must retrieve the sensed information from the other sensors, and then process it and check if some conditional rule applies in order to generate the appropriate event. Therefore, it depends on the way the information is retrieved from the sensors.

**Figure 4.**
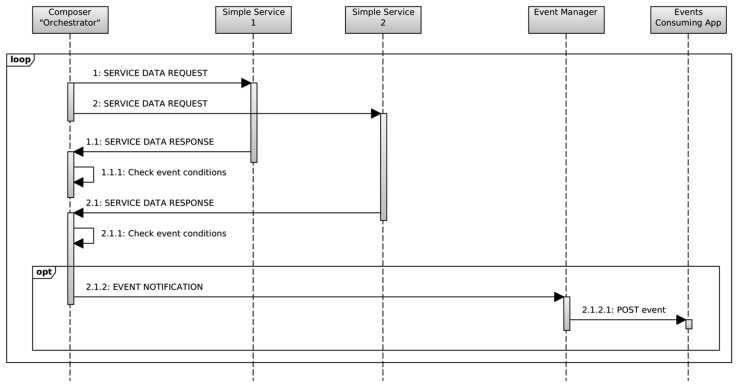
Event generation from a composed event source.

In our case the sensors are accessed by polling, so the *Orchestrator* periodically sends a request message to the registered services in order to get the new sensed data, as seen in the messages labeled as 1.X and 2.X in Figure 4. And then, as explained before, if the processing of the retrieved data implies the generation of an event, the *Orchestrator* follows the same behavior as any other event source, sending the same type of message directly to the *Event manager* as seen in Figure 4.

## 4. Results and Discussion

The model described in the previous section has been tested in a real scenario within the WoO project framework. For the purpose of this article, the use case evaluated here corresponds to an open smart neighborhood where the WSN is deployed in a house to provide the proper home automation through web services.

The sensors used during the tests were SunSPOT programmable motes. These sensors run on the ARM architecture, and have an integrated radio support in the 2.4 GHz band using IEEE 802.15.4. They are also equipped with a sensor board capable of providing temperature, luminosity and acceleration readings, along with the possibility of being interfaced with external sensors.

Indeed, one sensor used in these tests was a LV-MaxSonar-EZ0 used as a proximity sensor to detect possible undesired presences in the house. This sensor was virtually represented in the gateway.

Another sensor was programmed to virtualize a heat detector used for fire detection. In this case, the event service was composed using the temperature service offered by other sensors deployed in the network. Thereby this sensor can reuse other temperature services already deployed in any network to create a new event source for fire detection taking as a reference the operational specifications recommended by the European Normative EN 54-5:2000 [16].

In the test scenario there were three basic temperature sensors, the proximity sensor and the fire sensor, which also provided a mean temperature and was planned to serve as an in-network alarm dispatcher. Regarding the gateway, it was running on an Asus EB1033 PC using Ubuntu 12.04 LTS. The computer was connected to the WSN using a SunSPOT base station—a basic SunSPOT device without any kind of sensor board or battery—, using the abovementioned IEEE 802.15.4 radio interface. The gateway itself was a service integrated in an Enterprise Service Bus (ESB) platform using Fuse ESB Enterprise 7.1.

There are three main components in the gateway service. The first one is related to the nSOM middleware. It is in charge of discovering the services exposed by the devices in the WSN, and creating a virtual representation of them by exposing the access to their resources as REST services. It is also in charge of translating any message received from the WSN to the corresponding REST service, and reversely translating any REST invocation to the proper WSN message.

The second one is a registry that dynamically stores all the related information to the services discovered in the WSN, including the events sources and consumers. The registry was implemented using a MySQL 5.5 database server.

Last but not least, there is an event manager which is responsible for handling the events detected in the WSN and dispatching them to the list of registered consumers using the REST approach described in the proposal. The whole system has been integrated with other WoO partners' contributions to the open smart neighborhood scenario.

### 4.1. Measurements for Virtualizing an Existing Event Source

As explained in the proposal, the first idea of virtualization came from giving a virtual representation of an existing event source. There were two main measurements of interest: amount of time required to have a virtual representation of the event source, and time needed to notify the occurrence of an event using the virtual representation.

#### 4.1.1. Time to Virtual Representation of an Existing Event Source

As explained before, the time required to have a virtual representation of an existing event source as a REST service depends on three independent times:

(1) $$t_{\text{simple}\;\text{virtualization}}=t_{\text{discovery}}+t_{\text{registering}}+t_{\text{deploying}}$$

In Equation (1), *discovery time* involves the transmission time from the sensor to the gateway whenever the service is announced (passive discovery) or queried (active discovery); *registering time* involves the time required for storing in the database the information relevant to the service to be virtually represented, as the event source presented here; finally, *deploying time* involves the time required to expose the virtualized representation of the event source as a REST service.

**Figure 5.**
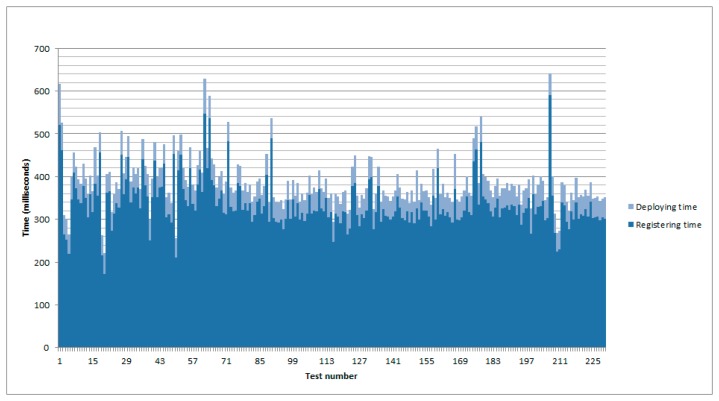
Time required getting a virtual representation of an existing event source in the gateway.

Discovery time is outside the scope of this paper, as it depends on other issues like the time required for the transmission of the messages between the nodes and the gateway. The environmental conditions have also an influence in the transmission, and therefore in discovery time. For the other two, Figure 5 shows the relation between both of them after 230 tests.

As it can be watched in the previously mentioned figure, the most time consuming task is precisely the one required to register the information of the event source in the database. Indeed, the total time required to have a virtual representation of the event source has a maximum value of 641 milliseconds and a minimum of 221, with an average value of 384.63 milliseconds and a standard deviation of 59.09. Another remarkable piece of information is the time required to register the event source; it shows a maximum value of 590 milliseconds and a minimum of 221 with an average one of 336.2 milliseconds and a standard deviation of 56.54. This represents between a 77.83% and a 92.04% of the total time.

#### 4.1.2. Time to Subscribe the Gateway to an Existing Event Source

**Figure 6.**
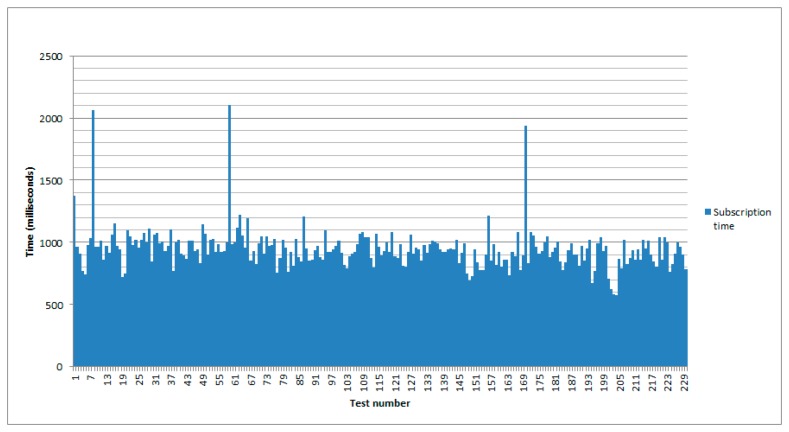
Time to subscribe the gateway to an event source.

Although it is not directly related to the virtualization itself, another interesting measurement is the required one to subscribe the gateway to the event source in the WSN. The process is quite simple: once the gateway is aware of the existence of an event source, it tries to subscribe to it. Figure 6 shows the results after 230 tests. As for the results, the maximum time for subscription was 2104 milliseconds and the minimum was 578. The average mean was 948.63 and the standard deviation was 167.54 milliseconds.

Taking a look at Figure 6 shows how there are a few times where the required time is almost the double of the usual. This is due to communication issues.

### 4.2. Measurements for Composing a New Virtual Event Source

The periods of time required to have the virtual representation of the composed virtual event source can be expressed as in Equation (2):

(2) $$t_{\text{composed}\;\text{virtualization}}=t_{\text{simple}\;\text{virtualization}}+t_{\text{composing}\;\text{event}\;\text{source}}$$

In the test scenario, the sensor that was composing the new virtual event source was programmed to request and retrieve information from the already deployed temperature sensors that were available at the moment of its own deployment. This means that any other temperature sensor that was turned on after the composing sensor was not taken into account for the composed event service.

For test purposes the composing sensor did not require a minimum number of temperature sensors in order to provide either the average temperature service or the fire event service. Obviously, this can be modified if required by the scenario where it is going to be used.

The fire detection was conducted following the guidelines of [16]. This standard determines the response of a fire alarm based on heat detectors as the result of an increase on the air temperature, and the response time is determined by the speed of the increase. Thus, for the virtual sensor the operation is quite simple: it just checks periodically the registered temperature services, and compares a set of consecutive readings in order to calculate the approximate speed of change in the temperature. Consequently, when it detects an increase on the temperature at a rate in the ranges determined by the standard, it generates a new event.

**Figure 7.**
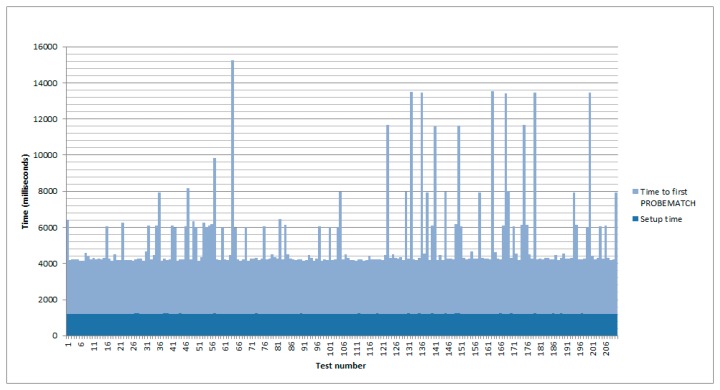
Time to event source composition.

Before that, the time period required to have a new virtual sensor deployed in the network was tested. In Figure 7 we have the results after 210 tests. The lower value, in blue, corresponds to the setup time of the device. It is the time used to deploy all the nSOM components, just before sending the PROBE message to locate the required services. As it can be observed, it is almost constant.

On the other hand, the timespan spent from the delivery of the PROBE message to the reception of the first PROBEMATCH, which defines the actual moment when the new virtualized event source is created, has also been considered. As expected, this second value is more irregular, because it depends on the transmission of both messages, and even in a small network there can be interferences.

When considering the total time, a maximum of 15,235 milliseconds, a minimum of 4142, with a mean of 5229.12 and a standard deviation of 2118.72, are obtained as the most representative values.

**Figure 8.**
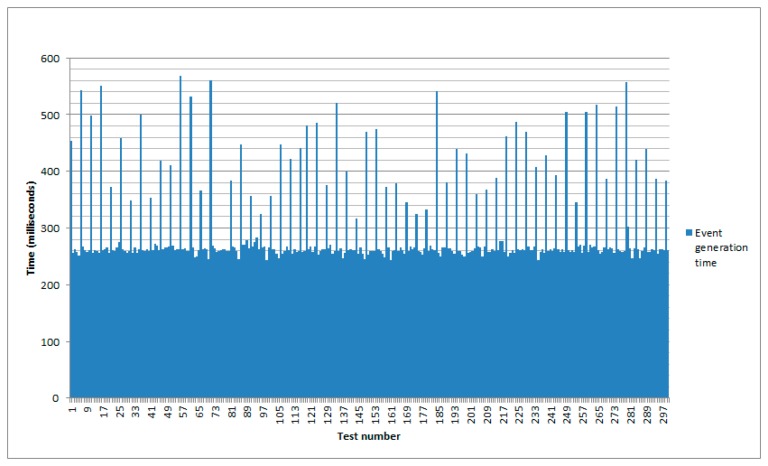
Time to generate an event.

Regarding event generation, the composed sensor sends a request periodically containing the current temperature value in the registered temperature sensors. The response is asynchronous, so the detection of a fire condition depends on the time required to retrieve the information from the implied temperature sensor and the lapse since the request was sent. Figure 8 shows the results for this part of the system after 300 tests.

For this test the maximum time required to generate an event once a fire condition has been detected in one of the nodes has been of 569 milliseconds, with a minimum of 243. The average mean has been of 295.05 milliseconds and the standard deviation 75.37 milliseconds.

This data, along with the previous ones, show what seems to be a periodical extra delay in the actions that require wireless communications between the devices involved both actions. This might be due to the routing protocol used by the devices, that is, the SunSPOT, which is an improved version of AODV called LQI. In this routing protocol the routes between two nodes in the network are discovered on demand, and stored for a predefined interval, so it makes sense that this extra time is precisely due to the extra amount of messages required to update the routing tables.

### 4.3. Measurements for Event Dispatching in the Gateway

**Figure 9.**
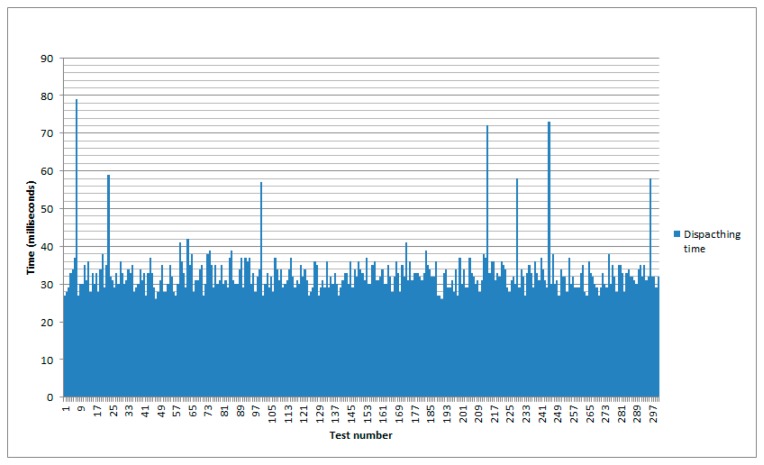
Time to dispatch an event from the gateway.

Another datum of interest is the time required for an event to be dispatched from the gateway once it has been received from the WSN. This time is measured from the moment an event is notified to the gateway to the time the POST message is delivered to the proper subscribers. Figure 9 shows the results of this after 300 tests.

In this case the maximum time has been of 79 milliseconds and a minimum of 26. The average mean has been of 32.78 milliseconds and the standard deviation 6.03 milliseconds.

### 4.4. Discussion

The results by themselves only show how the system behaves, but not if the performance is efficient or poor. In order to answer that question other conditions must be considered. As explained before, this work has been developed in the framework of the WoO project. This project has some requirements regarding the response time of a service, especially for events. In the WoO deliverable that defines the requirements of the system there is a strict condition regarding the system time response: it shall not exceed 1 min. So, when considering the results it can be affirmed that the proposed virtualization complies with the requirements of the system.

Although we have not planned to strictly follow EN 54-5, this standard also establishes some limits that can be used to measure the response of our composed approach to a fire detector. Considering the operating characteristics of the SunSPOT devices, we can consider them what [16] refers as a class A detector. For a class A1, the normative requires a response time between 1 min and 4 min and 20 s. Again, the results of the tests in both situations are below the requirements, so it can be concluded that the proposed virtualization model complies also with this requirement.

Some features of the deployment can be enhanced. The usage of a SQL database, for instance, is responsible for the longest delay in the virtualization of an existing event source. In order to save time in the registration part of the system, other structured data storage should be considered.

There is also another issue that can be observed in the actions where a wireless communication is required. In the event generation, for instance, it is quite easy to see that from time to time there is an increase in the time required. This extra amount of time is due to communication issues, and as explained in the previous section, this seems to be related to the update of the routing tables.

Another usual question is about the usage of REST for an event driven architecture. While REST is an effective a way of transferring the representation of the state of some resource, it does not make compulsory the usage of a specific protocol, although it was born from HTTP. So the final idea about this matter is that the REST paradigm can be followed by some other protocols, even those which provide a guarantee of delivery.

## 5. Conclusions

The communication protocol used by the WSN nodes, mentioned in the previous sections, is part of the nSOM middleware and enables its components to interoperate with each other. It is not standard, however, but it is quite similar to COAP as they both observe the ROA principles and avoid increasing the network or the node computational overhead significantly. Therefore, a significant improvement should be replacing the nSOM protocol by COAP, as the latter is a standard one recommended in several IoT based solutions. Nonetheless, COAP messages should be embedded straight to MAC layer frames, similar to the proposal in [17], to counter the negative impact on the performance of the COAP protocol stack, normally handled over TCP/IP.

Another significant issue that would leverage system interoperability is the pervasive use of semantic annotations, complying with well-accepted ontologies. One of the most overwhelming activities when integrating solutions from different stakeholders is mapping each one's information models. The system components must interchange semantically annotated data complying with a reduced set of well-accepted ontologies to overcome such drawbacks. Thus, providing the recipient with enough knowledge for unambiguous data processing is granted.

Regarding service composition, it should be kept in mind that it is one of the proposed design patterns for Service Oriented Architectures [18]. As an *Orchestrator* has been designed and developed, the importance of orchestration and choreography should be acknowledged, as they are two typical, well-established ways to design a service composition [19].

In the proposed model the orchestration concept has been selected, as it requires no extra capabilities in the already deployed basic sensors. A basic set of rules (capable of creating composed services for obtaining the average mean of a set of measurements, and creating new events when the measurements of the sensors go beyond certain threshold) has been defined. An additional rule has been added to the set so the composed service can also generate an event when the rate of change in the measurements exceeds some value.

The intention of this paper is proving that even constrained devices can be capable of composing some basic composed services, but the extension of this set of rules, allowing the creation of more complex services using any set event sources and/or other non-event service providers, remains as a task to be fully explored.

It is interesting to consider other works, as the HERMES proposal [20], to extend and improve the capabilities of our middleware proposal in the event management area. Although our interest in the virtualization goes beyond the basic aggregation, there are some interesting innovations in the publish/subscribe systems that can be of use to improve the results shown in this paper [21].

Considering again [19], the other typical way of composing a service is the choreography. Choreography implies that the sensors which have already been or are going to be deployed have also certain additional capabilities. However, unlike the orchestrator, the only responsibility of the choreographer is sharing some instruction sets to the service providers that are going to participate in the composed service. Even so, the exploration of choreography techniques applicable to the virtualization of WSNs is an interesting challenge.

Finally, there is also some work to be done with the communications. Some ideas that were proposed in the HERMES middleware [20] can be explored for their integration in nSOM. Also, communications can be synchronized in some scenarios [22], opening another path for exploration.

## Figures and Tables

**Table 1.** Event manager resources description.

**URI**	**Method**	**Description**
sources	POST	Register a new source.
	GET	Retrieve the list of registered sources.
sources/{sourceID}	GET	Retrieve the information of “sourceID”.
	PUT	Update the information of “sourceID”.
	DELETE	Remove the registered entry of “sourceID”.
subscribers	POST	Register a new subscriber.
	GET	Retrieve a list of registered subscribers.
subscribers/{subscriberID}	GET	Retrieve the information of “subscriberID”.
	PUT	Update the information of “subscriberID”.
	DELETE	Remove the registered entry of “subscriberID”.
